# Application of Platelet-Rich Plasma in Gynaecologic Disorders: A Scoping Review

**DOI:** 10.3390/jcm14165832

**Published:** 2025-08-18

**Authors:** Nadia Willison, Fariba Behnia-Willison, Pouria Aryan, Zahra Ali Padhani, Negin Mirzaei Damabi, Tran Nguyen, Johnny Yi, Rituparna Dutta, Derek Abbott

**Affiliations:** 1 FBW Gynaecology Plus, Ashford, SA 5035, Australia; fariba.behnia-willison@adelaide.edu.au (F.B.-W.); pouria.aryan@adelaide.edu.au (P.A.); tran.nguyen@adelaide.edu.au (T.N.); rituparnadutta1987@gmail.com (R.D.); 2 Discipline of Biomedical Engineering, School of EME, The University of Adelaide, Adelaide, SA 5005, Australia; derek.abbott@adelaide.edu.au; 3 School of Public Health, Faculty of Health and Medical Sciences, University of Adelaide, Adelaide, SA 5000, Australia; zahraali.padhani@adelaide.edu.au (Z.A.P.); negin.mirzaeidamabi@adelaide.edu.au (N.M.D.); 4 Robinson Research Institute, Faculty of Health and Medical Sciences, University of Adelaide, Adelaide, SA 5006, Australia; 5 Departments of Medical and Surgical Gynecology, Mayo Clinic, Phoenix, AZ 85054, USA; yi.johnny@mayo.edu

**Keywords:** regenerative medicine, platelet rich plasma, gynaecological conditions, vulvar lichen sclerosus, urinary incontinence, pelvic floor

## Abstract

Platelet-rich plasma (PRP) therapy is a non-invasive, autologous treatment with regenerative potential in gynaecology beyond fertility applications. This review evaluates PRP in non-fertility-related gynaecological conditions affecting women’s quality of life (QoL). **Methods:** Following PRISMA-ScR guidelines, we searched Embase, CINAHL, Web of Science, Scopus, CENTRAL, and MEDLINE for studies on PRP in conditions such as vulvar lichen sclerosus (VLS), vulvovaginal atrophy (VVA), sexual dysfunction (SD), stress urinary incontinence (SUI), and interstitial cystitis/bladder pain syndrome (IC/BPS). Of 3660 records screened, 43 studies (randomised controlled trials, quasi-experimental, cohort, and case series) were included. **Results:** PRP improved symptoms and QoL in several conditions, particularly VLS and SD, and was generally well tolerated with minor adverse effects (e.g., injection-site pain, transient discomfort). Evidence for abnormal uterine bleeding (AUB) and pelvic organ prolapse (POP) was inconclusive. Considerable heterogeneity in preparation protocols and outcome measures limited cross-study comparison. **Conclusions:** PRP shows promise as a minimally invasive therapy for certain gynaecological conditions. Standardisation of preparation and administration, along with large-scale RCTs, is needed to determine long-term efficacy and safety.

## 1. Introduction

Platelet-rich plasma (PRP) is a minimally invasive, non-hormonal therapy designed to support tissue repair, healing, and regeneration. Derived from the patient’s blood, PRP is a concentrated platelet-rich substance, with platelets essential for clotting and wound healing [1]. As an autologous therapy, it carries minimal risk of side effects and immunological reactions.

**Figure 1:**
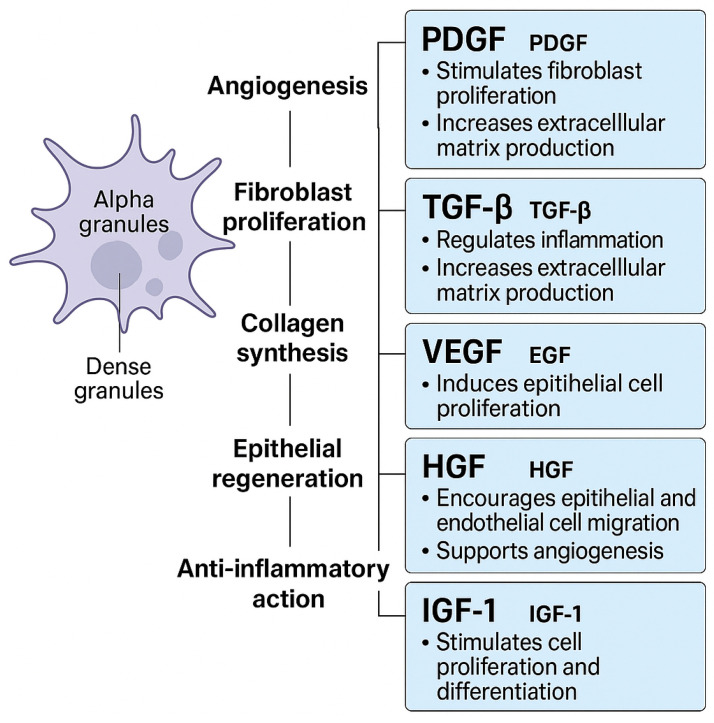
Key growth factors in platelet-rich plasma (PRP) and their biological roles. Platelets store these factors in alpha granules, releasing them upon activation to promote angiogenesis, fibroblast proliferation, collagen synthesis, epithelial regeneration, and anti-inflammatory effects. Shown here are PDGF, TGF-β, VEGF, EGF, HGF, FGF, and IGF-1, each contributing to tissue repair and healing through overlapping and synergistic pathways.

Although its precise mechanism remains unclear, PRP is thought to activate platelets, releasing various bioactive molecules. These molecules facilitate cell migration, proliferation, differentiation, angiogenesis, clearance of tissue debris, and tissue regeneration [2]. Recent clinical and experimental studies have demonstrated PRP’s ability to enhance wound healing, reduce inflammation, and improve postoperative tissue outcomes in both human and animal surgical models [3,4]. PRP is rich in seven key growth factors (GFs): PDGF, TGF-β, VEGF, EGF, HGF, fibroblast growth factor, and IGF-1 [5,6,7,8]. These GFs enhance healing by attracting stem cells to the fibrin matrix, initiating tissue repair and regeneration. They also suppress cytokine responses, reducing inflammation at the application site [9]. Additionally, the GFs recruit macrophages to remove cellular debris and promote new epithelial tissue formation [10]. They also stimulate fibroblast proliferation, migration, and colony formation, aiding scar repair. PRP may also contain white blood cells that contribute to tissue regeneration through their antimicrobial properties [9] (see Figure 1).

The application of PRP extends to orthopaedics, dentistry, dermatology, and cosmetic surgery to promote tissue repair, homeostasis, minimise scarring, enhance bone grafts, and treat tendonitis [11,12,13]. There is growing interest in its use in gynaecological conditions, particularly for vulvar and vaginal tissue repair and pelvic floor disorders (PFDs).

PRP is extracted, separated, and concentrated from the patient’s blood before being injected into the repair site. Upon activation, platelets release bioactive molecules that stimulate tissue regeneration. Studies show PRP improves sexual dysfunction (SD) by reducing vaginal dryness, atrophy, and laxity [14,15,16]. It has also shown potential in enhancing endometrial thickness and function in managing Asherman’s syndrome (AS) [13]. PRP therapy has also improved sexual function and quality of life (QoL) in women with lichen sclerosus [17,18], and successfully treated vulvovaginal atrophy, alleviating symptoms like vaginal burning, pain, and dryness [19].

PRP’s effectiveness in treating gynaecological conditions has been reported in cervical ectopy [20], vulvar dystrophy [21], vulvar cancer reconstructive surgery [20,21]. Also, PRP has been explored for urogenital disorders [22] and reproductive medicine, particularly to enhance endometrial and ovarian function [23,24].

While PRP’s role in fertility treatment is well established, this review uniquely examines its regenerative potential in treating non-fertility gynaecological conditions that impact women’s QoL. To date, no comprehensive review has explored PRP’s applications beyond fertility. This review focuses on conditions like AS, intrauterine adhesions (IUA), interstitial cystitis (IC), female genital mutilation (FGM), stress urinary incontinence (SUI), vulval lichen sclerosus (VLS), SD, and vulvovaginal atrophy (VVA). By analysing clinical outcomes, patient satisfaction, side effects, and research gaps, it highlights PRP’s therapeutic potential and proposes future research directions to advance its clinical applications in gynaecology.

## 2. Materials and Methods

We conducted a scoping review following the JBI guidelines for reporting results [25]. The protocol for this review is registered with The Open Science Framework (OSF) at https://doi.org/10.17605/OSF.IO/MF8XW (accessed on 21 August 2024).

### 2.1. Eligibility Criteria

We included experimental studies (RCTs and quasi-experimental) and observational studies (prospective and retrospective cohort studies and case studies). Eligible studies focused on PRP for gynaecological conditions, including VLS, SUI, SD, POP, FGM, AS, IC/BPS, vaginal atrophy, genitourinary syndrome of menopause, dyspareunia, IUA, fistula, and bacterial cystitis. Eligible populations included cisgender women of reproductive age, postmenopausal cisgender women, and transgender women. Outcomes of interest were symptom reduction, vaginal scarring, urinary incontinence, quality of life, sexual function, IUA grade, menstrual cycle changes, and treatment duration. Only studies published in English were included.

### 2.2. Exclusion Criteria

We excluded studies that did not address the gynaecological conditions specified in the eligibility criteria, including studies focused on fertility, ovarian PRP, caesarean section scars, or endometriosis.

Research involving platelet-rich fibrin (PRF), assisted reproductive technology (ART), mesh, or laser was omitted, together with animal studies and research involving men. We also excluded cross-sectional studies, systematic reviews, scoping reviews, grey literature, editorials, conference abstracts, commentaries, and letters to the editor.

### 2.3. Search Strategy and Study Selection

A search strategy was developed using MeSH terms and keywords related to PRP, women, and gynaecological conditions. The final search was conducted on 30 September 2024.

The search strategy was applied to the following electronic databases: Medline, EMBASE, CENTRAL (Cochrane Library), Scopus, CINAHL, and Web of Science. All identified studies were imported to the Covidence software [26] for de-duplication. Four people (N.W., Z.A., N.I., P.A.) independently screened titles, abstracts, and full texts for eligibility. Reference lists of relevant studies were also manually searched for additional articles not captured in the database search. Discrepancies were resolved by an additional person (F.B.W.).

### 2.4. Data Extraction and Analysis

After screening, data extraction was performed using Covidence software [26]. Four people (N.W., Z.A., N.I., P.A.) independently extracted data on study characteristics (author, year, title, design, setting), participant details, disease condition(s), intervention, PRP preparation, frequency of administration, patient follow-up, and outcome measures. Any disagreements between the two primary reviewers were resolved through discussion; if consensus could not be reached, the matter was adjudicated by an independent third reviewer who was not involved in the initial screening or data extraction. Descriptive and thematic analyses were conducted, categorised by gynaecological conditions.

A scoping review approach was chosen in accordance with PRISMA-ScR guidelines to map the breadth and characteristics of available evidence on PRP in non-fertility-related gynaecological conditions. This methodology was deemed appropriate given the substantial heterogeneity in study designs, PRP preparation protocols, and outcome measures, which precluded meaningful quantitative synthesis.

## 3. Results

**Figure 2:**
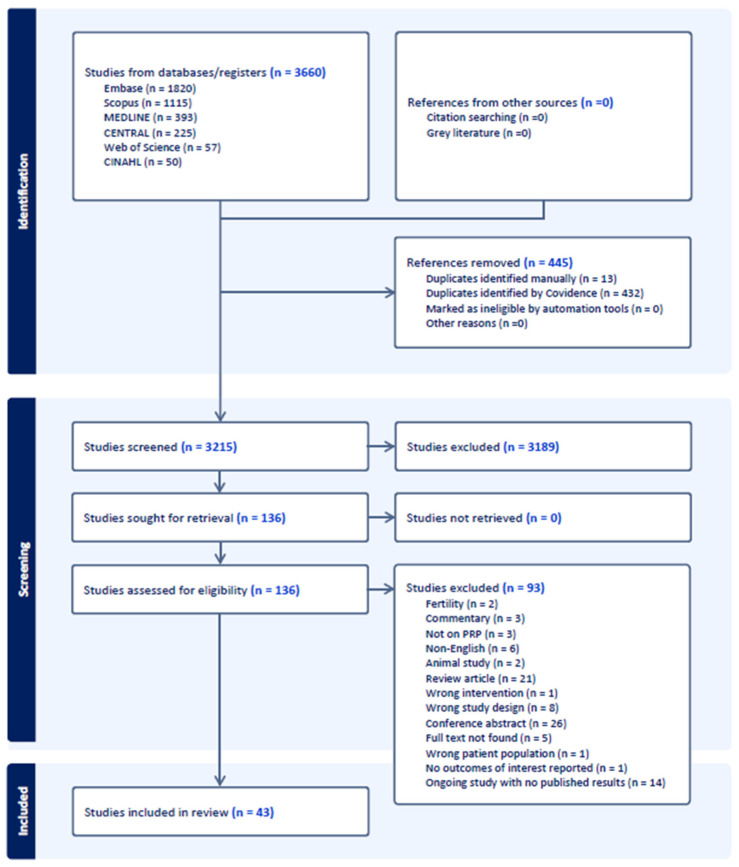
PRISMA flow diagram.

The initial search identified 3850 studies. After de-duplication, 3332 studies underwent title and abstract screening, followed by full-text screening of 143 studies. Of these, 97 studies were excluded, with reasons detailed in Figure 2. The final review included 46 studies: two on vulvovaginal atrophy, one on anterior POP, one on vaginal mesh exposure, three on SD, one on AUB, one on vesicovaginal fistula, and one on recurrent bacterial cystitis. Additionally, four studies examined AS, three on IUA, six on VLS, seven on IC/BPS, two on recurrent UTI, one on both rUTI and IC/BPS, eight on SUI, three on FGM and one on PFD. The characteristics of the included studies are summarised in Table 1.

### 3.1. Vulvovaginal Atrophy (VVA)

Two studies reported on VVA [19,27]. Omar et al. [27] conducted a multi-arm RCT with 45 sexually active female cancer survivors experiencing VVA symptoms due to cancer treatment. Participants were randomised into three groups: Group A received two submucosal vaginal PRP injections one month apart; Group B received two PRP injections plus non-crosslinked hyaluronic acid one month apart; and Group C applied topical vaginal hyaluronic acid gel three times weekly for two months. Groups A and B showed significant improvements in vaginal pH, fluid volume, and VHI scores. Group A had the greatest reduction in dyspareunia, while Group B showed superior improvement in vaginal dryness over Group C. Topical hyaluronic acid had no significant effect on vaginal elasticity. Injection-related pain was reported in all patients receiving PRP, with some reporting vaginal spotting.

Saleh et al. [19] conducted a pilot study on 47 postmenopausal women diagnosed with VVA (per VHI criteria) who received two PRP treatments one month apart. After one-month post-treatment, the Vulvovaginal Symptom Questionnaire (VSQ) showed significant improvements in sexual relationship, vaginal dryness, discharge, burning, pain, irritation, libido, dyspareunia, and dryness during sexual activity. No AEs were reported.

### 3.2. Anterior Pelvic Organ Prolapse (A-POP)

A prospective cohort study [28] assessed the use of autologous platelet gel (APG) in nine women undergoing anterior colporrhaphy for A-POP. APG was applied to the surgical site post-repair. A 6 mm biopsy from the anterior vaginal wall was taken before surgery and three months postoperatively to assess collagen content, which showed no significant change in pubocervical fascia collagen content (9.04 mg/mg w.w. vs. 9.58 mg/mg w.w., where w.w. refers to wet weight). The subjective failure rate, defined as the patient’s report of persistent or recurrent symptoms, was 12.5%, while the objective failure rate, based on clinical examination or imaging findings, was 66.7%, with a 12.5% reoperation rate.

### 3.3. Vaginal Vault Mesh Exposure (VVME)

A case series [29] involving three women reported VVME after laparoscopic abdominal sacral colpopexy (ASC) with concomitant hysterectomy. Patients presented with perineal pain, deep dyspareunia, and/or vaginal bleeding/discharge, with a history of dyspareunia and/or vaginal bleeding/discharge. All underwent endoscopic bipolar PlasmaKinetic resection (BPR), successfully removing the exposed mesh without residual mesh or vaginal perforations. PRP was injected along the resected margins and PRP gel applied to the surgical site. Follow-up at one year showed no recurrence of dyspareunia or POP, and all women had recovered sexual function.

### 3.4. Sexual Dysfunction (SD)

Three studies investigated PRP for SD [17,30,31]. Hamadani et al. [17] conducted a comparative interventional study with 20 women with SD and vaginal laxity undergoing posterior colpoperineorrhaphy, with half receiving PRP injections. Both groups showed significant improvements in sexual function, but PRP enhanced sexual interest, activity, and pleasure. No significant differences were found in sexual satisfaction, orgasm ability, or perceived importance of sex, suggesting PRP may target specific aspects of sexual function.

Sukgen et al. [30] performed a cross-sectional study with 52 women with SD who received four PRP injections to the anterior vaginal wall, spaced four weeks apart. Female Sexual Function Index (FSFI) scores increased from 13.61 to 27.88 at six months, with 50% achieving normal sexual function (FSFI ≥ 26). Significant improvements were also seen in the Female Genital Self-Image Scale and Female Sexual Distress Scale, with high patient satisfaction and no AEs.

A prospective cohort study by Dardeer et al. [31] found PRP injections into the anterior vaginal wall and clitoris improved FSFI scores across all domains (*p* < 0.05) in 45 women, including 29 with FGM/C. No AEs were reported, suggesting PRP may be a safe, minimally invasive, and effective treatment for SD in both FGM/C and non-FGM/C women.

### 3.5. Female Genital Mutilation/Cutting (FGM/C)

Three studies have explored the potential of PRP therapy for FGM/C. Manin et al. [32] presented a case study of a 35-year-old woman with FGM/C Type IIb who underwent clitoral reconstruction with PRP therapy, reporting complete pain relief and full healing two months post-procedure. Tognazzo et al. [2] documented a case study of five women with FGM/C Type III receiving PRP after reconstructive surgery, with all achieving complete healing by day 80, faster than the typical 90 days, alongside reduced postoperative pain and lower painkiller use. Dardeer et al. [31] conducted a prospective cohort study assessing PRP’s effects on sexual function in both FGM/C and non-FGM/C women, finding significant improvements in desire and arousal in both groups, suggesting PRP may enhance sexual well-being post-FGM/C.

### 3.6. Abnormal Uterine Bleeding (AUB)

A RCT [33] evaluated the effectiveness of intracavitary PRP therapy in patients with AUB. The study included 149 participants (PRP group: 74; control group: 75). Patients in the PRP group received intracavitary PRP injections following endometrial curettage, while the control group underwent endometrial curettage alone. Outcomes were evaluated three months post-treatment using transvaginal ultrasonography. The results showed no significant difference between the PRP and control groups in reducing endometrial thickness or bleeding, thereby concluding that PRP therapy did not provide additional benefits over endometrial curettage alone.

### 3.7. Vesicovaginal Fistula (VVF)

A prospective study [34] evaluated the use of PRP and platelet-rich fibrin glue (PRFG) for VVF repair in 12 patients. The treatment resulted in a 91.67% cure rate after a single session, with significant improvements in urinary incontinence and QoL scores. No complications or AEs were reported, suggesting that this technique is a promising alternative to traditional surgical methods.

### 3.8. Recurrent Bacterial Cystitis (RBC)

A RCT by Mirzaei, M., et al. [35] evaluated intravesical PRP instillation (administered weekly for four weeks) in 30 women with RBC. At 12 months, the PRP group had a significantly greater reduction in cystitis recurrences compared to placebo (3.66 ± 2.16 vs. 1.46 ± 1.59, *p* = 0.004) and significant improvement in ICIQ-OAB symptom scores (*p* ≤ 0.001), while the control group showed no significant change (*p* = 0.89). No AEs were reported up to 12 months, suggesting intravesical PRP instillation may be a safe and effective treatment for reducing recurrence and improving symptoms in women with RBC.

### 3.9. Asherman’s Syndrome (AS)

Four studies investigated PRP for AS [1,36,37,38]. Aghajanova et al. [1] evaluated the efficacy of instilling 0.5–1 mL PRP or saline into the uterus immediately after hysteroscopy, followed by oestrogen therapy. No significant change in endometrial thickness was observed between the PRP and saline groups. Javaheri et al. [36] conducted a non-RCT study with 30 women diagnosed with AS. After hysteroscopic adhesiolysis, participants received either 1 mL of PRP or no PRP instilled into the uterine cavity. Hysteroscopy 8-10 weeks later showed no differences in menstrual bleeding patterns or IUA stage between groups. A RCT by Naghshineh, E. et al. [37] randomised 60 AS patients to receive either hormonal therapy alone or PRP plus hormonal therapy post-hysteroscopy. Follow-up showed no differences in menstrual patterns or IUA grades I–III between groups.

Aghajanova et al. [38] reported two AS cases treated with intrauterine PRP. A 34-year-old with secondary amenorrhea showed modest endometrial thickening (3.8 to 5.4 mm) after standard treatment, with slight improvement post-PRP (4.8 to 5.0 mm). A 40-year-old with recurrent pregnancy loss showed greater endometrial thickening (3.3 to 6.7 mm) after PRP and achieved a viable pregnancy. PRP was well tolerated, with no AEs, and appeared to improve endometrial function, leading to successful conception and pregnancy.

### 3.10. Intrauterine Adhesions (IUAs)

Three studies evaluated PRP for IUAs [39,40,41]. An RCT by Guangwei et al. [39] compared autologous platelet gel (APG) a fibrin-based semi-solid product formed by activating PRP, with medical chitosan in 80 patients post-transcervical resection of adhesions. The APG group showed a lower recurrence rate of IUAs (21% vs. 49%) and improved adhesion scores, though platelet concentration had no effect. Shen et al. [41] conducted an RCT with 63 patients, showing that PRP combined with an intrauterine balloon resulted in a greater reduction in adhesion scores compared to the balloon alone (AFS score reduction: 7 vs. 6). A retrospective cohort study by Peng et al. [40] found no significant differences in adhesion scores or chemical pregnancy rates among PRP, intrauterine balloon therapy, or their combination. These early findings suggest PRP may improve post-hysteroscopy outcomes, but further studies are needed to confirm its efficacy compared to standard treatments.

### 3.11. Vulvar Lichen Sclerosus (VLS)

Six studies investigated PRP for VLS [18,42,43,44,45,46]. A retrospective cohort study by Casabona et al. [18] reported significant QoL improvements in 72 women treated with PRP and fat grafting. Similarly, Medina Garrido et al. [42] found PRP reduced itching and soreness for up to a year, assessed using the Clinical Scoring System for VLS. Behnia-Willison et al. [43] found that after three PRP injections in 28 women with steroid-resistant VLS, over 80% discontinued steroids, and more than half experienced symptom relief at 12 months. A case report by Franić et al. [44] described a 38-year-old woman who showed significant improvements in vaginal symptoms and sexual health after two PRP treatments.

Recent studies have explored innovative methods to enhance and monitor PRP therapy for VLS. Tedesco et al. (2021) [45] used video thermography in six women, showing that thermal imaging detected temperature changes linked to symptom relief. A follow-up study [47] with 94 participants found gender-specific responses to PRP, with women reporting greater relief from pain and burning, and men showing improvements in sexual discomfort.

Boero et al. (2024) [46] conducted a self-controlled pilot study in 50 women with corticosteroid-refractory VLS, administering three sessions of autologous PRP injections. At six-month follow-up, all patients reported treatment satisfaction. Symptom severity scores (itching, burning, dyspareunia) decreased significantly, alongside improvements in FSFI, HADS, and SF-12 scores. The need for maintenance topical corticosteroids was reduced by 42%, and all patients showed enhanced vulvar elasticity and colour on clinical examination.

### 3.12. Interstitial Cystitis/Bladder Pain Syndrome (IC/BPS)

Seven studies investigated PRP for IC/BPS [48,49,50,51,52,53]. An observational case–control pilot study [48] with 15 women found PRP injections resulted in significant pain reduction, lower symptom scores, and increased bladder capacity. A prospective cohort study [52] with 40 patients report symptom relief, improved inflammatory markers, and no AEs. A comparative study [51] found that lower, repeated PRP doses provided the best outcomes for 63 patients. Another study [49] showed PRP’s positive effects on cell health and bladder barrier function.

Further studies highlight PRP’s benefits over traditional therapies. A 2023 retrospective cohort study [54] showed PRP and Botulinum Toxin A (BoNT-A) both relieved symptoms, with PRP having fewer side effects. A 2024 prospective cohort study [50] identified specific cytokine levels in PRP that could predict treatment success, allowing for personalised therapy. El Hefnawy, A.S. et al. [55] found that six weekly PRP instillations in 21 women with IC/BPS led to significant pain reduction (VAS decreased by 50.1%, *p* = 0.001) and symptom improvement; 80% of patients responded positively, suggesting that repeated PRP instillations may offer a safe and effective treatment for IC/BPS treatments.

### 3.13. Recurrent Urinary Tract Infection (rUTI)

Two prospective cohort studies investigated PRP for rUTIs. Lee and Kuo [56] compared intravesical PRP injections (monthly for four months) with continuous antibiotics for three months in 63 women. PRP significantly reduced rUTI frequency, with a 51.5% success rate, similar to the 48% success rate with antibiotics. Women with higher baseline voiding efficiency showed better outcomes with PRP. However, the study called for further research to assess the long-term effectiveness of PRP compared to antibiotics. Jiang et al. [53] reported a 63.6% success rate in 22 women with rUTIs following repeat PRP injections. Bladder biopsies showed increased expression of proteins linked to cell proliferation and differentiation, suggesting PRP may improve bladder health and reduce UTI recurrence in persistent infections.

### 3.14. Stress Urinary Incontinence (SUI)

The included studies comprised two randomised controlled trials, one comparative cohort study, two prospective interventional studies, and two case series investigated PRP for SUI. Behnia-Willison et al. [57] combined PRP with fractional carbon dioxide laser therapy in 62 women, with two-thirds reporting significant symptom improvement within three months, and most maintaining relief for up to two years without serious AEs. Daneshpajooh et al. [58] compared periurethral PRP injections with the standard midurethral sling procedure, showing 70% improvement with PRP, while the sling procedure achieved full recovery in 80% of women. The study suggested additional PRP doses could potentially enhance its efficacy in this context. Grigoriadis et al. [59] found PRP significantly reduced symptoms compared to placebo, with about one-third of the 50 participants reporting little or no leakage after six months. Saraluck et al. [60] administered two PRP injections with a one-month interval combined with pelvic floor muscle training (PFMT) in 60 women, resulting in 90% reporting at least a 50% reduction in symptoms, significantly outperforming PFMT alone. Long et al. [61] observed substantial symptom relief in 20 women following PRP injections, with no reported AEs.

Ashton et al. [62] conducted a randomised, placebo-controlled trial assessing the efficacy of a single 5 mL PRP injection into the anterior vaginal wall at the mid-urethra in 50 women with stress-predominant urinary incontinence. At six months, composite treatment success—defined as both subjective improvement and a negative cough stress test—was not significantly different between the PRP and placebo groups (16% vs. 4.5%). No major adverse events were reported, and minor AEs were comparable across groups. Compared to earlier observational studies reporting higher success with multiple PRP treatments, this trial suggests that a single injection may be insufficient, highlighting the need for further research into optimised protocols.

Meghna et al. [63] presented a case series on PRP therapy for cervical ectropion, recurrent candidiasis, and stress urinary incontinence (SUI). PRP was prepared using a two-step centrifugation method and administered locally. Two doses led to symptom resolution and healing of chronic cervicitis in a case of cervical ectropion. A postmenopausal woman with recurrent candidiasis, unresponsive to antifungals, achieved complete remission after two intravaginal PRP injections. For SUI, three monthly PRP treatments improved the vaginal health score from 10 to 18 and reduced incontinence episodes. No adverse events were reported. These findings suggest PRP is a safe, minimally invasive treatment that can provide substantial symptom relief and improve QoL for women with SUI.

### 3.15. Pelvic Floor Dysfunction (PFD)

For PFD, a single RCT by Moegni et al. [64] conducted between November 2016 and July 2019 across 21 health facilities in Jakarta, Indonesia, assessed whether PRP could aid in healing levator ani muscle (LAM) trauma from childbirth, a common cause of PFD. The prospective, single-blind study included first-time pregnant women who received PRP or placebo injections during perineorrhaphy after delivery. Out of 240 initially enrolled women, 58 completed the study. The results showed no significant differences between the PRP and placebo groups in LAM strength or levator hiatal area, indicating PRP did not enhance muscle recovery in this context.

Table 1 is condensed to emphasise protocol, effectiveness, and side effects. Detailed PRP preparation parameters are summarised separately in Section 3.16; NR = not reported; ↑ = increase; ↓ = decrease. Table 2 summarises the outcome instruments used across studies, aligned with Table 1. Only the most reported tools are listed.

**Table 1:** Preparation of PRP in the included studies.

Condition	Study (Year)	Design/Sample Size	PRP Protocol (Brief)	Effectiveness Summary	Side Effects
Vulvovaginal Atrophy (VVA)	Omar et al. (2023) [27]	RCT, *n* = 45	2 injections, 4 wks apart; ± hyaluronic acid	↑ VHI; improved pH and fluid volume; ↓ dyspareunia (PRP alone); ↓ dryness (PRP+HA vs. HA gel)	Injection pain; mild spotting
Vulvovaginal Atrophy (VVA)	Saleh et al. (2022) [19]	Pilot, *n* = 47	2 injections, 4 wks apart	VSQ: improvements in dryness, discharge, burning, pain, irritation, libido, dyspareunia	NR
Sexual Dysfunction (SD)	Al-Hamadani et al. (2019) [17]	Comparative, *n* = 20	2 injections (intraop + 4 wks)	Improved sexual interest, activity, pleasure; no significant change in satisfaction, orgasm ability	NR
Sexual Dysfunction (SD)	Sukgen et al. (2019) [30]	Prospective, *n* = 52	4 injections, 4 wks apart, anterior vaginal wall	FSFI ↑ (13.6 → 27.9); 50% reached normal FSFI ≥ 26; improved genital self-image and distress scores	NR
Sexual Dysfunction (SD)	Dardeer et al. (2022) [31]	Prospective cohort, *n* = 45 (incl. FGM)	1 injection, anterior vaginal wall + clitoris	FSFI improved in all domains; benefit in both FGM and non-FGM groups	NR
Female Genital Mutilation (FGM/C)	Manin et al. (2022) [32]	Case report, *n* = 1	1 injection post-reconstruction	Complete pain relief; full healing by 2 mo	NR
Female Genital Mutilation (FGM/C)	Tognazzo et al. (2023) [65]	Case series, *n* = 5	1 injection post-reconstruction	Complete healing by day 80; reduced pain and analgesic use	NR
Vulvar Lichen Sclerosus (VLS)	Casabona et al. (2023) [18]	Retrospective cohort, *n* = 72	Median 4 injections over 3 yrs	QoL improvement; symptom relief; lesion resolution	NR
Vulvar Lichen Sclerosus (VLS)	Medina Garrido et al. (2023) [42]	Prospective, *n* = 20	3 injections, 4–6 wks apart	↓ itching and soreness up to 1 yr	NR
Vulvar Lichen Sclerosus (VLS)	Behnia-Willison et al. (2016) [43]	Prospective, *n* = 28	3 injections, 4–6 wks apart	>80% discontinued steroids; >50% symptom relief at 12 mo	Mild pain
Vulvar Lichen Sclerosus (VLS)	Franić et al. (2018) [44]	Case report, *n* = 1	2 injections, 8 wks apart	Improved vaginal symptoms, sexual health	NR
Vulvar Lichen Sclerosus (VLS)	Tedesco et al. (2021) [45]	Prospective, *n* = 6	3 injections, 2 wks apart	Thermography: temperature changes linked to symptom relief	NR
Vulvar Lichen Sclerosus (VLS)	Boero et al. (2024) [46]	Self-controlled pilot, *n* = 50	3 injections, 4–6 wks apart	↓ itching, burning, dyspareunia; improved FSFI, HADS, SF-12; ↓ steroid need	NR
IC/BPS	Jhang et al. (2019) [48]	Case-control pilot, *n* = 15	4 intravesical injections, 4 wks apart	Pain ↓; ↑ bladder capacity; improved symptom scores	NR
IC/BPS	Jiang et al. (2020) [52]	Prospective, *n* = 40	4 intravesical injections, 4 wks apart	Symptom relief; improved urinary functional proteins	NR
IC/BPS	Jiang et al. (2022) [51]	Comparative, *n* = 63	Single vs. multiple injections; dose comparison	Lower repeated doses had best outcomes	NR
IC/BPS	Jhang et al. (2022) [49]	Prospective, *n* = 19	4 injections, 4 wks apart	↑ urothelial proliferation; improved cytoskeleton and barrier proteins	NR
IC/BPS	Jhang et al. (2023) [50]	Prospective, *n* = 40	4 injections, 4 wks apart	TNF-α in PRP associated with outcomes (predictive)	NR
IC/BPS	Jhang et al. (2023) [54]	Comparative, *n* = 60	4 PRP injections vs. BoNT-A	Both effective; PRP with fewer adverse events	NR
IC/BPS	El Hefnawy et al. (2024) [55]	Prospective, *n* = 21	6 instillations, weekly	VAS pain ↓ 50%; 80% responders	NR
Recurrent UTI (rUTI)	Lee & Kuo (2023) [56]	Comparative, *n* = 63	4 intravesical injections, monthly	51.5% success; similar to continuous antibiotics	NR
Recurrent UTI (rUTI)	Jiang et al. (2021) [53]	Prospective, *n* = 22	4 intravesical injections, 4 wks apart	63.6% success; ↑ proliferation/repair markers in biopsies	NR
Abnormal Uterine Bleeding (AUB)	Turan et al. (2018) [33]	RCT, *n* = 149	Single intracavitary PRP post-curettage	No significant benefit vs. curettage alone (thickness/bleeding)	NR
Anterior POP (A-POP)	Einarsson et al. (2009) [28]	Prospective feasibility, *n* = 9	Topical autologous platelet gel	No sig. change in collagen content; high objective failure; 12.5% reop.	NR
Vaginal Vault Mesh Exposure (VVME)	Castellani et al. (2017) [29]	Case series, *n* = 3	BPR mesh resection + PRP injection/gel	No recurrence of dyspareunia/POP at 12 mo; sexual function restored	NR
Vesicovaginal Fistula (VVF)	Shirvan et al. (2013) [34]	Prospective, *n* = 12	PRP injection + PRF glue	91.7% cure after single session; QoL improved	NR
Asherman’s Syndrome (AS)	Aghajanova et al. (2021) [1]	RCT, *n* = NS	0.5–1 mL intrauterine PRP vs. saline	No sig. change in endometrial thickness	NR
Asherman’s Syndrome (AS)	Javaheri et al. (2020) [36]	Non-RCT, *n* = 30	1 mL PRP post-adhesiolysis vs. none	No differences in bleeding pattern or IUA stage	NR
Asherman’s Syndrome (AS)	Naghshineh et al. (2023) [37]	RCT, *n* = 60	PRP + hormones vs. hormones post-hysteroscopy	No differences in menstrual pattern or IUA grades I–III	NR
Asherman’s Syndrome (AS)	Aghajanova et al. (2018) [38]	Case series, *n* = 2	Two intrauterine instillations	Case 1: modest thickening; Case 2: viable pregnancy achieved	NR
Intrauterine Adhesions (IUAs)	Guangwei et al. (2023) [39]	RCT, *n* = 80	Autologous platelet gel vs. chitosan post-TCRA	↓ IUA recurrence (21% vs. 49%); improved adhesion scores	NR
Intrauterine Adhesions (IUAs)	Shen et al. (2022) [41]	RCT, *n* = 63	PRP + balloon vs. balloon alone	Greater AFS score reduction with PRP	NR
Intrauterine Adhesions (IUAs)	Peng et al. (2020) [40]	Retrospective, *n* = 97	PRP vs. balloon vs. combo	No sig. differences in adhesion score or chemical pregnancy	NR
Stress Urinary Incontinence (SUI)	Behnia-Willison et al. (2020) [57]	Prospective, *n* = 62	3 periurethral injections, 4–6 wks apart + CO_2_ laser	≈66% improved within 3 mo; relief maintained up to 2 yrs	NR
Stress Urinary Incontinence (SUI)	Daneshpajooh et al. (2021) [58]	RCT, *n* = 40	Single periurethral PRP vs. sling	PRP 70% improved; sling 80% cured	NR
Stress Urinary Incontinence (SUI)	Grigoriadis et al. (2024) [59]	RCT, *n* = 50	2 injections, 4–6 wks apart	↓ symptoms; ~⅓ little/no leakage at 6 mo	NR
Stress Urinary Incontinence (SUI)	Saraluck et al. (2024) [60]	RCT, *n* = 60	2 injections, 4 wks apart + PFMT	≥50% symptom reduction in 90% of participants	NR
Stress Urinary Incontinence (SUI)	Long et al. (2021) [61]	Prospective, *n* = 20	3 injections, 4 wks apart	Substantial symptom relief	NR
Stress Urinary Incontinence (SUI)	Ashton et al. (2024) [62]	RCT, *n* = 50	Single anterior vaginal wall injection	No sig. difference vs. placebo (16% vs. 4.5%)	NR
Stress Urinary Incontinence (SUI)	Meghna et al. (2024) [63]	Case series, *n* = 3	2–3 injections, 4 wks apart	Symptom resolution and VHI improvement	NR
Pelvic Floor Dysfunction (PFD)	Moegni et al. (2022) [64]	RCT, *n* = 58	Single injection during perineorrhaphy	No significant difference in LAM strength/hiatal area	NR

**Table 2:** Effectiveness assessment tools used across PRP studies (by condition).

Condition	Assessment Tools Used	What They Measure (Summary)	Representative Studies
Vulvovaginal Atrophy (VVA)	VHI, VSQ, vaginal pH/fluids	Vaginal health (elasticity, pH, moisture), symptom burden	Omar 2023 [27]; Saleh 2022 [19]
Sexual Dysfunction (SD)	FSFI, FGSIS/FGSI, FSD Distress	Sexual function domains; genital self-image; distress	Sukgen 2019 [30]; Dardeer 2022 [31]
Female Genital Mutilation (FGM/C)	FSFI, wound healing/time to heal, analgesic use	Sexual function; postoperative recovery	Tognazzo 2023 [65]; Manin 2022 [32]; Dardeer 2022 [31]
Vulvar Lichen Sclerosus (VLS)	Clinical LS scores, FSFI, QoL scales, thermography	Pruritus, pain, dyspareunia, sexual function; thermal changes	Behnia-Willison 2016 [43]; Medina Garrido 2023 [42]; Tedesco 2021/2022 [45,47]; Boero 2024 [46]
IC/BPS	VAS, ICSI/ICPI, bladder capacity, biomarkers	Pain and symptom severity; bladder function; inflammatory signatures	Jhang 2019/2022/2023 [48,49,50,51,54]; Jiang 2020/2022 [49,51,52]; El Hefnawy 2024 [55]
Recurrent UTI (rUTI)	Recurrence episodes, voiding efficiency, urothelial biomarkers	Infection frequency; lower urinary tract function; epithelial integrity	Lee & Kuo 2023 [56]; Jiang 2021 [53]
AUB	TVUS endometrial thickness; bleeding pattern	Structural change; symptom improvement	Turan 2018 [33]
A-POP	Collagen content (biopsy); failure rates	Tissue composition; objective/subjective failure	Einarsson 2009 [28]
VVME	Recurrence of exposure; dyspareunia; sexual function	Mesh complications; sexual outcomes	Castellani 2017 [29]
VVF	Cure/closure rate; QoL; continence	Fistula resolution; patient-reported outcomes	Shirvan 2013 [34]
AS	Endometrial thickness; menstrual pattern; IUA stage	Endometrial recovery; adhesion severity	Aghajanova 2021 [1]; Javaheri 2020 [36]; Naghshineh 2023 [37]
IUAs	AFS/adhesion score; recurrence; chemical pregnancy	Adhesion burden and recurrence; fertility proxy	Guangwei 2023 [39]; Shen 2022 [41]; Peng 2020 [40]
SUI	ICIQ-SF; 1-h pad test; cough stress test; patient-reported	Symptom severity; objective leakage; clinical stress response	Behnia-Willison 2020 [57]; Daneshpajooh 2021 [58]; Grigoriadis 2024 [59]; Saraluck 2024 [60]; Long 2021 [61]; Ashton 2024 [62]
PFD	LAM strength; levator hiatus area (imaging)	Pelvic floor muscle recovery postpartum	Moegni 2022 [64]

Table 2 summarises the outcome instruments used across studies, aligned with Table 1. Only the most reported tools are listed.

### 3.16. PRP Preparation Methods Included Studies

Given the substantial variability in PRP preparation techniques observed across studies, we summarised the reported protocols to highlight commonalities, differences, and their potential implications for clinical outcomes. Variability was evident across almost all preparation parameters, including whole blood volume collected, centrifugation settings, platelet concentration achieved, leucocyte content, and activation strategy. Such heterogeneity—also discussed in the Discussion—is a key factor limiting cross-study comparability.

General Process

Most protocols began with venepuncture to collect autologous whole blood into anticoagulant-containing tubes, followed by either a single or double centrifugation (“spin”) to separate plasma and concentrate platelets. The platelet-rich fraction was aspirated and either injected directly or activated before administration.

Centrifugation Protocols

- Single-spin methods (e.g., Saleh et al., 2022 [19]: RegenKit^®^ A-PRP, 1500 g for 5 min; Sukgen et al., 2019 [30]: 3200 rpm for 8 min) were faster and simpler, reducing platelet loss, but typically yielded lower platelet concentrations.
- Double-spin methods (e.g., Omar et al., 2023 [27]; Grigoriadis et al., 2024 [59]: ~9× baseline platelet concentration) achieved higher enrichment but were more complex and carried a greater risk of premature activation if handling was suboptimal.
- Blood Volume and PRP Yield
- Drawn volumes ranged from 5 mL (Aghajanova et al., 2018 [38]) to 100 mL (Jiang et al., 2022 [51]), producing final yields of 1–10 mL PRP depending on technique and target concentration.

Leucocyte Content

- Leucocyte-rich PRP (LR-PRP): Generated when the buffy coat layer was included (e.g., Sukgen et al., 2019 [30]), potentially offering antimicrobial benefits and a stronger inflammatory phase, but sometimes increasing post-injection discomfort.
- Pure PRP (P-PRP): Prepared to minimise leucocytes (e.g., Moegni et al., 2022 [64]), favouring reduced inflammatory response—potentially beneficial for chronic inflammatory conditions such as VLS.

Activation Methods

- Chemical activation: Calcium chloride (e.g., Sukgen et al., 2019 [30]), calcium gluconate (e.g., Omar et al., 2023 [27]), or thrombin-rich serum (e.g., Castellani et al., 2017 [29]) to trigger immediate growth factor release, which may shorten bioactive availability.
- No exogenous activation: Used in several studies (e.g., Behnia-Willison et al., 2016 [43]) to allow in vivo clotting, enabling slower, potentially prolonged release.

Commercial Closed Systems

Systems such as RegenKit^®^ (Regen Lab SA, Le Mont-sur-Lausanne, Switzerland), Magellan^®^ (Arteriocyte Medical Systems, Inc., Hopkinton, MA, USA), and Arthrex ACP (Arthrex GmbH, Munich, Germany) offered standardisation and sterility—often with regulatory clearance—but were costlier and less adaptable for protocol modification.

Reasons for Variation

Protocol differences reflected the target condition (e.g., mucosal injection, intravesical instillation, surgical coating), desired biological profile (pro-inflammatory vs. anti-inflammatory), device and consumable availability, jurisdictional regulations, and operator or institutional preference.

Reporting Gaps

Several studies omitted key parameters such as platelet concentration, leucocyte content, activation strategy, or centrifugation details. Classification frameworks (e.g., PAW, Mishra, Dohan Ehrenfest) were rarely applied. Standardising these elements would improve reproducibility, facilitate meta-analysis, and support the development of evidence-based preparation guidelines (Dohan Ehrenfest et al., 2009 [66]).

## 4. Discussion

This scoping review highlights the growing interest in PRP as a therapeutic option for non-fertility-related gynaecological conditions. Across the 13 conditions reviewed, PRP demonstrated varying efficacy, with notable improvements in SD, VLS, and SUI. While conventional vaginal reconstructive surgery has been performed for decades, PRP may represent a potential non-surgical alternative or adjunct for pre-, intra-, and postoperative management of PFD [61,67,68]. Studies by Al-Hamadani et al. [17] and Dardeer et al. [31] reported significant improvements in sexual desire, arousal, and lubrication after PRP injections. Similarly, Behnia-Willison et al. [43] and Casabona et al. [18] found significant symptom relief and lesion resolution in VLS patients. For AUB and A-POP, however, the results were mixed, with some studies showing minimal benefits compared to standard therapies, highlighting the need for further research to confirm PRP’s effectiveness in these areas.

The combination of PRP with other treatments showed promoting results, suggesting a potential synergistic effect. For example, PRP combined with non-crosslinked hyaluronic acid for VVA treatment [27] showed superior vaginal hydration compared to hyaluronic acid alone. Similarly, Behnia-Willison et al. [57] found that combining PRP with fractional CO_2_ laser therapy enhanced symptom relief in SUI. These findings suggest that PRP may be most effective as an adjunctive therapy, though further studies are needed to confirm this.

Across the studies, PRP was well tolerated, with minimal AEs reported. The most common AEs were transient pain at the injection site, vaginal spotting, and mild discomfort, especially with vulvovaginal injections. No serious AEs were reported, suggesting PRP is a relatively safe treatment for gynaecological conditions. However, the lack of long-term follow-up in most studies prevents conclusions regarding the durability of its effects and its long-term safety.

### 4.1. Limitations

A key limitation of the current literature is the substantial variability in study designs, PRP preparation protocols, and outcome measures, making it difficult to draw definitive conclusions on efficacy and safety. Preparation techniques ranged from single-spin (e.g., Dardeer et al. 2022 [31]) to double-spin centrifugation (e.g., Omar et al. 2023 [27]), with some studies employing standardised commercial systems such as RegenKit^®^ and Magellan. However, technical details were often underreported. Other inconsistencies included blood draw volumes (5–100 mL) producing PRP yields of 1–10 mL, platelet concentrations ranging from 1.6× to 9× baseline (e.g., Grigoriadis et al. 2024 [59]), and marked variation in injection protocols, frequency (single or multiple sessions at 1–6 week intervals), and activation strategies (e.g., calcium gluconate, thrombin-rich serum, or in vivo clotting).

Most included studies did not report or stratify outcomes by menopausal status, limiting the ability to assess potential differences in PRP effectiveness between premenopausal and postmenopausal women. Although some conditions examined (e.g., vulvovaginal atrophy) predominantly affect postmenopausal women, hormonal influences on vascularisation, collagen synthesis, and tissue healing may also be relevant across other age groups. Future research should stratify and analyse results by menopausal status to clarify these potential differences.

The absence of a standardised protocol for PRP preparation and administration remains a major challenge. As outlined in Section 3.16, variability in commercial devices, centrifugation parameters, anticoagulant use, and the inclusion or exclusion of leukocytes or activators results in inconsistent PRP formulations. Outcomes are further influenced by patient-specific factors such as age, hormonal status, and overall health. Cross-disciplinary use of PRP across orthopaedics, dermatology, dentistry, and gynaecology has led to specialty-specific adaptations rather than a unified protocol, complicating direct comparison between studies and hindering the development of universally accepted best practices.

In addition, the durability of PRP’s therapeutic effects remains uncertain. Follow-up periods across the included studies varied widely—from weeks to over 12 months—limiting insight into long-term outcomes, particularly for chronic conditions such as vulvar lichen sclerosus and stress urinary incontinence. High-quality studies with extended and standardised follow-up are needed to determine the sustainability of treatment benefits.

Finally, practical barriers to clinical implementation must be acknowledged. PRP requires trained personnel for blood collection, centrifugation, and condition-specific preparation, together with access to specialised equipment and appropriate infrastructure. Such resources, along with regulatory approvals, may not be available in all healthcare settings—particularly within female pelvic health—potentially limiting standardisation, reproducibility, and broader adoption.

### 4.2. Strengths

This review is, to our knowledge, the first to comprehensively map the current evidence on platelet-rich plasma (PRP) for a broad range of non-fertility-related gynecological conditions, integrating both qualitative findings and available quantitative outcome data. By synthesising clinical evidence alongside the biological roles of the key growth factors present in PRP, we provide a clinically relevant and mechanistically informed overview. The inclusion of diverse study designs, varied patient populations, and multiple outcome measures offers a wide perspective on current practice and highlights areas for future research. This dual focus on clinical outcomes and mechanistic context aims to support evidence-based decision-making and guide the development of standardised PRP protocols in women’s pelvic health. Across the 43 included studies, sample sizes ranged from 5 to 200 participants (median ~48), with follow-up periods varying from 4 weeks to 12 months. Most studies (approximately 65%) reported positive symptomatic or quality-of-life improvements following PRP therapy, with adverse effects generally mild and transient.

## 5. Conclusions

PRP may offer a safe, minimally invasive therapeutic option for selected gynaecological disorders such as VLS, SD, and VA, with preliminary evidence suggesting symptom improvement in patients unresponsive to conventional therapies. However, current findings are largely based on small-scale or heterogeneous studies, and robust evidence from well-designed RCTs is still lacking—particularly for conditions like AUB and A-POP, where data remain sparse.

Future research should prioritise large, methodologically rigorous RCTs, standardisation of PRP preparation and administration protocols, and evaluation of combination therapies. Extended follow-up is also required to clarify the durability of PRP’s effects, especially in chronic conditions such as VLS and SUI.

## Data Availability

Data sharing is not applicable to this article as no new datasets were generated or analysed during the current study. All data analysed in this review were derived from previously published studies, which are cited within the manuscript. Further information regarding data sources can be found in the references section.

## Abbreviations

The following abbreviations are used in this manuscript:

A-POP	Anterior Pelvic Organ Prolapse
AEs	Adverse Effects
AFS	American Fertility Society (score)
ART	Assisted Reproductive Technology
AS	Asherman’s Syndrome
ATP	Adenosine Triphosphate
ADP	Adenosine Diphosphate
AUB	Abnormal Uterine Bleeding
BoNT-A	Botulinum Toxin A
CO_2_	Carbon Dioxide
EGF	Epidermal Growth Factor
ECGF	Endothelial Cell Growth Factor
FGF	Fibroblast Growth Factor
FGM/C	Female Genital Mutilation/Cutting
FGSI/FGSIS	Female Genital Self-Image Scale/Score
FSFI	Female Sexual Function Index
FSD	Female Sexual Dysfunction
GDNF	Glial Cell Line-Derived Neurotrophic Factor
HADS	Hospital Anxiety and Depression Scale
HGF	Hepatocyte Growth Factor
IC/BPS	Interstitial Cystitis/Bladder Pain Syndrome
ICPI	Interstitial Cystitis Problem Index
ICIQ-SF	International Consultation on Incontinence Questionnaire—Short Form
ICSI	Interstitial Cystitis Symptom Index
IGF-1	Insulin-Like Growth Factor-1
IUA(s)	Intrauterine Adhesion(s)
LAM	Levator Ani Muscle
LS	Lichen Sclerosus
NR	Not Reported
NS	Not Specified
OSF	Open Science Framework
PAW	Platelet Activation White Blood Cell Classification
PDAF	Platelet-Derived Angiogenesis Factor
PDGF	Platelet-Derived Growth Factor
PFD	Pelvic Floor Dysfunction
PF4	Platelet Factor 4
PFMT	Pelvic Floor Muscle Training
POP	Pelvic Organ Prolapse
PRF	Platelet-Rich Fibrin
PRISMA-ScR	Preferred Reporting Items for Systematic Reviews and Meta-Analyses Extension for Scoping Reviews
PRP	Platelet-Rich Plasma
QoL	Quality of Life
RBC	Recurrent Bacterial Cystitis
rUTI(s)	Recurrent Urinary Tract Infection(s)
SD	Sexual Dysfunction
SF-12	12-Item Short Form Survey
SUI	Stress Urinary Incontinence
TCRA	Transcervical Resection of Adhesion
TGF-β	Transforming Growth Factor Beta
TVUS	Transvaginal Ultrasound
VA	Vulvovaginal Atrophy
VAS	Visual Analogue Scale
VEGF	Vascular Endothelial Growth Factor
VHI	Vaginal Health Index
VLS	Vulvar Lichen Sclerosus
VVA	Vulvovaginal Atrophy
VVME	Vaginal Vault Mesh Exposure
VVF	Vesicovaginal Fistula
VSQ	Vulvovaginal Symptom Questionnaire
